# A Framework for Instantaneous Driver Drowsiness Detection Based on Improved HOG Features and Naïve Bayesian Classification

**DOI:** 10.3390/brainsci11020240

**Published:** 2021-02-14

**Authors:** Samy Bakheet, Ayoub Al-Hamadi

**Affiliations:** 1 Department of Information Technology, Faculty of Computers and Information, Sohag University, P. O. Box 82533 Sohag, Egypt; 2 Institute for Information Technology and Communications (IIKT), Otto-von-Guericke University Magdeburg, 39106 Magdeburg, Germany; ayoub.al-hamadi@ovgu.de

**Keywords:** driver drowsiness detection, HOG features, shifted orientations, NB classification, NTHU-DDD dataset

## Abstract

Due to their high distinctiveness, robustness to illumination and simple computation, Histogram of Oriented Gradient (HOG) features have attracted much attention and achieved remarkable success in many computer vision tasks. In this paper, an innovative framework for driver drowsiness detection is proposed, where an adaptive descriptor that possesses the virtue of distinctiveness, robustness and compactness is formed from an improved version of HOG features based on binarized histograms of shifted orientations. The final HOG descriptor generated from binarized HOG features is fed to the trained Naïve Bayes (NB) classifier to make the final driver drowsiness determination. Experimental results on the publicly available NTHU-DDD dataset verify that the proposed framework has the potential to be a strong contender for several state-of-the-art baselines, by achieving a competitive detection accuracy of 85.62%, without loss of efficiency or stability.

## 1. Introduction

Globally, an average of 3200 persons die each day around the world due to road traffic crashes (RTCs). It is estimated that driver-related dangerous behaviors such as drowsiness, drug and alcohol use, inexperience and psychological stress, are contributing factors in the vast majority of these crashes, and driver drowsiness is the most commonly reported reason among non-performance errors that accounted for such crashes [1]. For example, in the U.S., the National Highway Traffic Safety Administration (NHTSA) reported that drowsy driving was responsible for an estimated 3662 fatal crashes and 4121 fatalities from 2011 to 2015, which corresponds to 2.4 percent of all fatal crashes and 2.5 percent of all crash fatalities recorded in the U.S. during the same period [2].

The relationship between driver drowsiness and crash risk has been investigated extensively in numerous studies, with the purpose of identifying and quantifying the increased risk. For instance, in [3], Williamson et al. provides clear evidence for sleep homeostatic effects producing impaired performance and accidents. Additionally, it has been reported that drowsy drivers are more likely to be involved in sleep-related crashes or near-crashes than attentive drivers by nearly 4–6 times. Recently, in a case-control study of heavy-vehicle drivers, Stevenson et al. [4] found that cumulative sleep deprivation or sleep debt can also increase the likelihood of having a serious crash. Furthermore, in [5], Li et al. stated that drowsiness can seriously impair the ability to drive properly, as people find it difficult to maintain their attention on the task. In other words, lack of sleep can make drivers less alert and affect their coordination, judgement and reaction time while driving.

Over the past few years, several approaches and techniques that significantly contribute to reducing road trauma have been developed, such as training drivers in improved fatigue-management practices—e.g., having the required rest breaks [6]. This depends on subjective measures consisting of self-assessment of one’s drowsiness levels. A relatively recent study [7] found that drivers have some ability to identify their current state of being drowsy and likelihood of falling asleep. Despite self-assessment of drowsiness being a relatively good initial coping mechanism, it is not able to get rid of drowsiness-related road trauma completely, so complementary warning and safety systems urgently need to be developed.

Technological innovations hold great potential to drive a considerable reduction in the number of both injuries and fatalities associated with road accidents, through alerting drivers to their drowsy state prior to accidents. For example, a study by Blommer et al. [8] has reported a significant improvement in driver reaction times in lane-departure scenario, when a warning signal is issued. The authors also concluded that the manner in which such warning signals are issued is shown to be not absolutely crucial. In other words, visual, auditory and haptic warnings are all equally operative.

Drowsiness detection methodologies found in the literature are generally categorized into three main categories, namely, vehicle-based measurements, physiological measurements and computer vision techniques. In traditional vehicle-based measurement approaches, sudden or large corrections in traveling directions are typically detected through calculating the values of a number of driving behavior metrics, such as deviations from lane position [9] and movements of the steering wheel [10]. An alternative approach for detecting changes in driver alertness level is to monitor and trace specific internal signals, such as heart rate variability [11] or brain activity [12]. However, due to the need for multiple sensors, physiological measurement approaches often turn out to be less practically feasible in the real world, compared to both vehicle-based and computer vision approachers [13]. In this context, it is worth mentioning that the great disadvantage with physiological sensors is that they are obtrusive and will thus never be used in a production vehicle.

Computer vision techniques based on artificial neural networks (ANNs) have been successfully (and still being) applied to many road safety problems (e.g., traffic safety analysis of toll plazas [14] and identifying behavioral changes among drivers [15]). These techniques have also emerged as leading architectures for many visual recognition tasks [16]. In the past few years, driver drowsiness detection has drawn great attention from the computer vision and object recognition community. In [17], Park et al. presented an automated system for driver drowsiness detection using three pre-trained deep neural networks (i.e., VGG-FaceNet, AlexNet and FlowImageNet) and two ensemble strategies (independently averaged architecture and feature-fused architecture) to classify each frame in an input video sequence as drowsy or not. In a similar vein, in [18], the authors proposed a driver drowsiness detection method using a 3D deep neural network along with a boosting framework for semi-supervised learning to improve supervised learning.

In [19], Rateb et al. presented a deep learning based approach for real-time drowsiness detection that can be monolithically integrated into android applications with a high accuracy rate. The primary contribution in this work involves the compression of a heavy baseline model to a lightweight model. Further, in this approach, a minimized network structure is designed to determine whether the driver is drowsy or not, depending on facial landmark (key point) detection. In [20], an eye blinking detection approach was presented using innovative color and texture segmentation algorithms, where the driver’s facial features are obtained by a facial segmentation and neural network-based algorithm. The obtained features are then utilized for iris tracking and eye blinking detection. In their method, each eye closure longer than 220 ms is identified as drowsy or asleep.

In [21], Pauly and Sankar presented a method for drowsiness detection based on traditional histogram of oriented gradient (HOG) features and support vector machines (SVMs) for blink detection. The method was validated on their own dataset, achieving an overall drowsiness detection accuracy of 91.6%, by comparing the prediction of the developed system with that of a human observer. Moreover, in [22], a face-monitoring based framework for drowsy driver detection was proposed, where a concise face texture descriptor is utilized to identify the most discriminant drowsiness features. Similarly, in [23], Singh et al. also proposed the use of HOG feature extraction and linear SVM classification to detect oncoming driver fatigue and issue a sufficiently early warning to help in preventing the accident. In this paper, the main contributions can be summarized as follows. First, a framework is developed for designing a vision-based system for instantaneous driver drowsiness detection. Secondly, we introduce an innovative feature descriptor depicting an improved version of HOG features based on binarized histograms of shifted orientations. The naïve Bayes (NB) classification model is then modified by adding the correlation between the data samples. This not only allows the NB algorithm to be a dependent hypothesis, but also enables the proposed framework to effectively tackle the problem of the classification of large-scale datasets. The remainder of this paper is structured as follows. In Section 2, we provide a detailed description of the proposed system for instantaneous driver drowsiness detection. Experimental evaluation results are then reported and discussed in Section 3. Finally, Section 4 provides concluding remarks with some thoughts for future work.

## 2. Proposed Architecture

**Figure 1:**
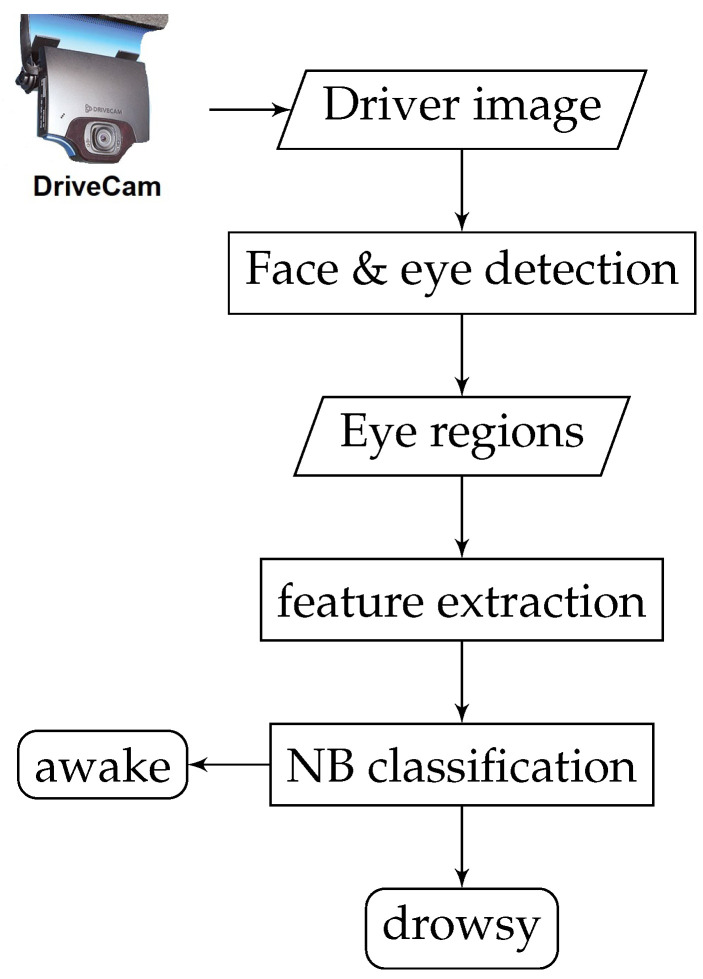
A functional block diagram of the proposed framework for driver drowsiness detection.

In this section, the details of the proposed framework for driver drowsiness detection are introduced. A functional block diagram for the key framework steps is depicted Figure 1. The brief explanation of the methodology for detecting driver drowsiness using the proposed framework is as follows. The input drive image captured by a dashboard mounted camera is initially preprocessed by applying adaptive contrast-limited histogram equalization for reducing the fluctuations in lighting intensity and thus enhancing the overall brightness and contrast in the image. Then, the driver’s face is detected by a cascaded adaBoost classifier based on Haar-like features [24]. For locating the eye-pair region, a simple and effective algorithm based on an improved active shape model (ASM) is applied. A set of potentially discriminative HOG features based on orientation-shifted histograms is extracted from the detected eye-pair regions, and finally fed into an NB classifier to predict the eye status. More details for each designed component in the detection framework are provided in the following subsections.

### 2.1. Image Preprocessing

Initially, the input driver image captured by an in-vehicle camera mounted on the dashboard is convolved with a 2D Gaussian blur filter over a 3×3 pixel neighborhood and uniform standard deviation of 0.5 to get rid of (or suppress) disturbing noises and unwanted background spots, while retaining spatially varying image structures. For light compensation, an adaptive contrast-limited histogram equalization algorithm [25,26] is then applied, with which each color channel is independently equalized to produce a better lighting-compensated image which further serves as input to the subsequent face-detection module. After the light compensation, the resolution of the light-compensated image can be reduced to increase the computational efficiency of the framework [27].

### 2.2. Eye Localization

**Figure 2:**
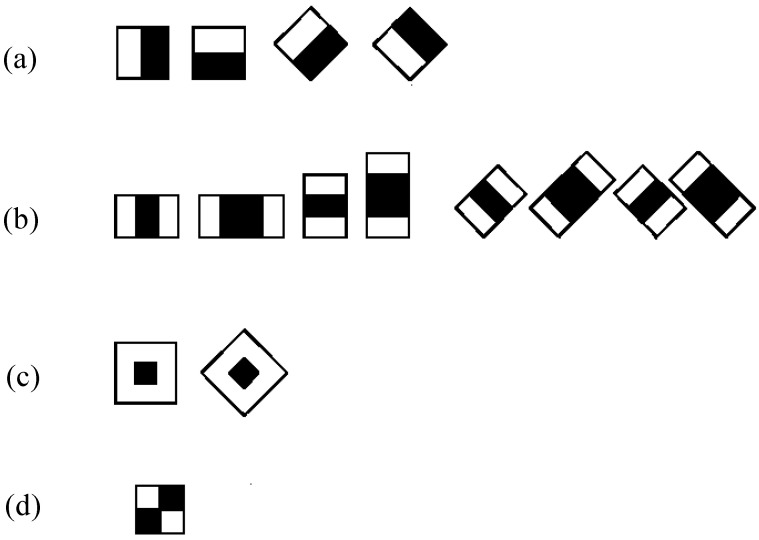
Extended Haar-like features: (**a**) edge features, (**b**) line features, (**c**) center-surrounded features and (**d**) a special diagonal line feature.

As mentioned earlier, the first and very crucial step in developing and implementing an effective framework for drowsiness detection involves face detection and eye-pair localization (i.e., regions of interest—ROIs), which aims at locating the positions of the driver’s eyes. In this work, a fast face detection algorithm was developed, where an improved cascaded adaBoost classifier based on an extended set of Haar-like features (see Figure 2) is utilized to automatically recognize the driver’s face. In this algorithm, as a face can be located at any position and scale in the input image, the compensated image is first split into a number of rectangular regions. As it can be seen in Figure 2, the employed features are defined as different arrangements of bright regions and dark regions. In each case, the feature value corresponds to the difference between the sum of pixel intensities within the bright regions and the sum of pixel intensities within the dark regions. Due to the rapid training pace of improved Haar-like features, they have great potential for real-time face detection.

**Figure 3:**
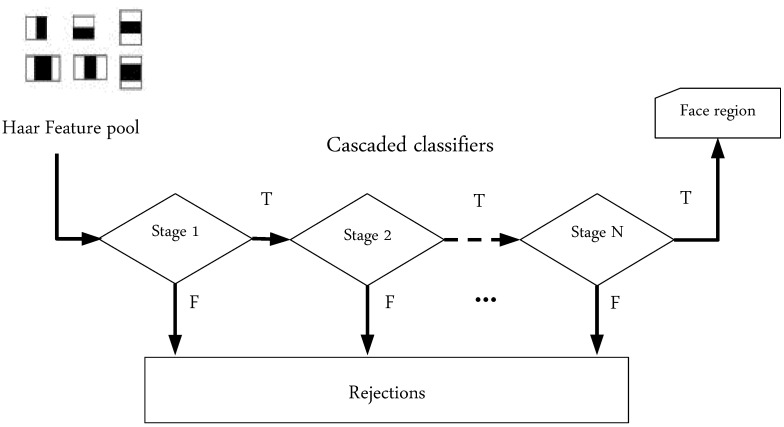
The cascade structure of adaBoost classifier for face detection.

A cascaded adaBoost classifier basically is a strong (nonlinear) classifier built upon an ensemble of several weak (linear) classifiers; each is trained using the adaBoost algorithm. The face region is found when a candidate sample percolates through the cascaded adaBoost classifier. Nearly all face samples are allowed to pass through, while non-face samples are rejected. Waterfall-type classification using the adaBoost algorithm for face detection is shown in Figure 3.

**Figure 4:**
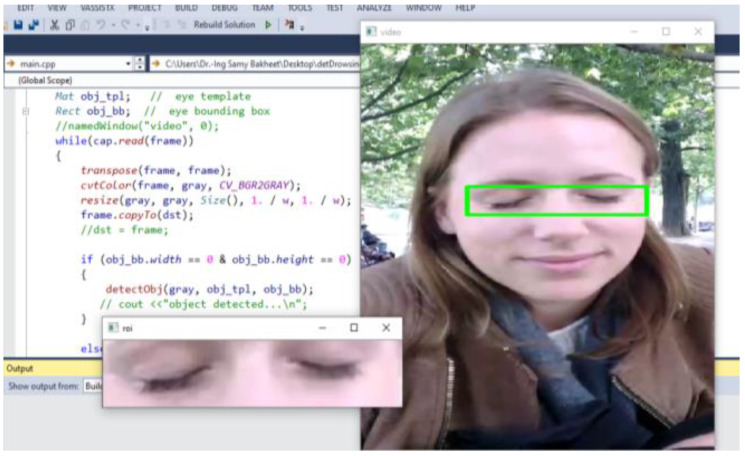
A sample snapshot of the resultant eye-pair region localization in our framework for driver drowsiness detection.

For detecting an eye-pair region, we use an improved active shape model (ASM) algorithm based on statistical learning models, which has the potential to extract relevant facial features rapidly and effectively. In this approach, the ultimate aim of active shapes is to match the model to a new image. To accomplish this goal, the ASM is trained on a set of specific points representing facial feature contours which has been manually labeled with facial feature interest points. Principal component analysis (PCA) is then performed to find out the main modes of variation in the training dataset. After establishing the ASM, the eye-pair regions in the face image are detected and localized, as shown in Figure 4. To reduce the difference between the model and the real contour, an iterative scheme using a cost function can be adopted to match models iteratively.

### 2.3. Feature Extraction

Due to their high descriptive power, robustness to illumination variation and simplicity and ease of implementation, HOG features originally initiated by N. Dalal and B. Triggs [28] have been extensively used (and still in use) in diverse domains of computer vision, such as face recognition, vehicle detection, video surveillance, image retrieval and disease diagnosis [29]. In this section, we show how to extract a modified variant of HOG features that resides in a relatively low-dimensional space and has significant discriminative power to properly characterize and quantify textures of eye and mouth regions for instantaneous driver drowsiness detection. In the HOG descriptor, the features are computed by taking orientation histograms of edge intensity in local eye regions. To achieve this objective, two fundamental computation units (i.e., cell and block) are locally defined. For each HOG feature, the block size is set to be 2×2 cells each of size 8×8 pixels, and blocks partially overlap—namely, each cell is covered by four blocks.

To extract the HOG features, the gradient and orientation values are first computed at each pixel location (x, y), by the application of the 1D centered point discrete derivative mask with the filter kernel [−1, 0, 1]. For this goal, we initialize by calculating the magnitude ρ(x, y) and direction γ(x, y) for each pixel value as follows.

(1) $$\begin{array}{ccc} \rho & = & \sqrt{L_{x}{\left(x,\;y\right)}^{2}+L_{y}{\left(x,\;y\right)}^{2}}\\ \gamma & = & \arctan\left(\frac{L_{y}\left(x,\;y\right)}{L_{x}\left(x,\;y\right)}\right) \end{array}$$

where $L_{x}$ and $L_{y}$ are the first-order Gaussian derivatives of the image patch luminance *I* in the *x* and *y* directions, respectively, which are computed at a scale of parameter σ (i.e., standard deviation) as follows.

(2) $$L_{\xi }=I*\frac{\partial }{\partial_{\xi }}\left(\frac{1}{2\pi \sigma^{2}}e^{-\left(x^{2}+y^{2}\right)/2\sigma^{2}}\right){|}_{\xi =x|y}$$

where ∗ denotes 2D discrete convolution. The gradient magnitude ρ of each pixel in the cell is then voted into a specified number of angular bins (e.g., 8 bins) according to the orientation of the pixel’s gradient. For every pixel in the orientation image, a histogram of orientations is built over a local spatial window (i.e., cell), such that the contribution of each pixel to an orientation bin is weighted by the gradient magnitude. More formally, the weight of each pixel, which is denoted by α, is computed as follows:

(3) $$\alpha =b+0.5-\frac{\gamma }{\pi }m$$

where *b* and *m* are the histogram bin to which γ belongs and the total number of bins in the histogram, respectively. To eliminate or reduce aliasing, we propose to update both values of two adjacent bins as follows:

(4) $$\tilde{\gamma }=\left(1-\alpha \right)\gamma ,\;\hat{\gamma }=\alpha \gamma$$

**Figure 5:**
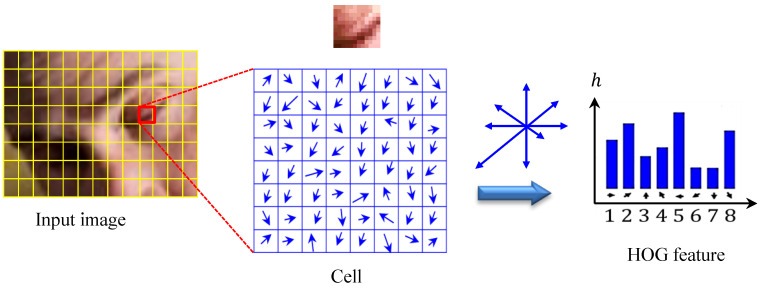
Extraction process of histogram of oriented gradient (HOG) features.

Then, the weighted votes $\tilde{\gamma }$ are accumulated into histogram bins over local spatial regions, so-called cells. The process to extract HOG features form a sample eye-pair image is shown in Figure 5.

**Figure 6:**
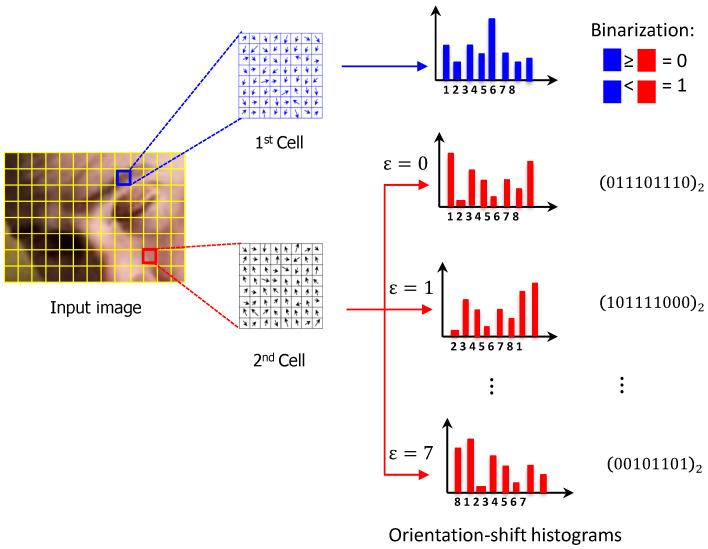
HOG feature extraction with a shift in the orientation.

In this work, we adopt an adaptive strategy to strengthen the description of HOG features, where similarities between image patches are utilized to capture the relatedness of local spatial regions. For this purpose, the orientation bins of the 8-bin HOG histogram created from a single cell are incrementally shifted by a factor ε(ε=0, 1,…, 7), resulting in a total of eight 8-bin histograms. Then, the binarized HOG feature quantities of two cell regions c1 and c2 are directly computed by comparing the size relationships of the 8-bin orientation-shifted histograms:

(5) $$b_{c1c2}\left(k,\;\varepsilon \right)=\left\{\begin{array}{cc} 1,\; & \mathrm{if}\;v_{c1}\left(k\right)\geq v_{c2}\left(\left(k+\varepsilon \right)\%8\right).\\ 0,\; & \mathrm{otherwise}. \end{array}\right.$$

where % denotes the modulo (division remainder) operator. It is worthy of pointing out that the extraction of HOG features based on binarized orientation-shifted histograms (see Figure 6) not only has potential to produce a more compact and robust version of HOG features, but also reduces greatly the computational time to a point compromising real-time execution. This, in turn, inevitably contributes greatly to faster and more accurate object detection.

**Figure 7:**
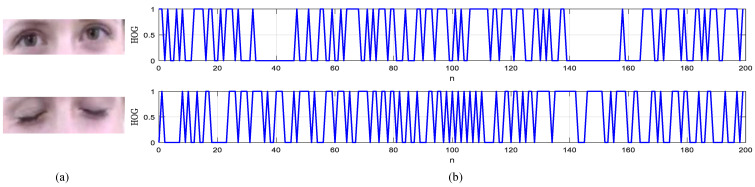
2D visualization plots for the improved HOG descriptor of the HOG features extracted from two eye region snapshots: (**a**) input eye region image and (**b**) binarized HOG descriptor based on orientation shift.

As an illustrative example, in Figure 7, we present 2D visualization plots for the developed HOG descriptor based on binarized orientation-shift HOG features extracted from two eye region snapshots.

### 2.4. Bayesian Feature Classification

**Figure 8:**
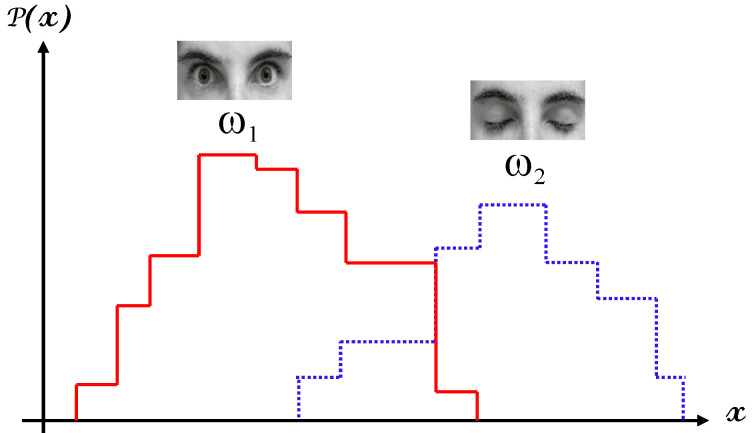
A histogram of feature values against their probability for two classes $\omega_{1}$ and $\omega_{2}$.

In this section, we describe in detail the classification module based on an NB algorithm in the proposed system for instantaneous driver drowsiness detection. Strictly speaking, NB is a simple probabilistic model [30] based on applying Bayes’ theorem [31] with strong independence assumptions among the attributes. Conventional probabilistic classifiers (e.g., NB) depend entirely on the conversion of data into probabilities for classification. As a representative example, in Figure 8, we show measurements of a given feature x for two classes $\omega_{1}$ and $\omega_{2}$. As can be observed in the figure, the members belonging to the first class tend to have larger values than those of the second class; however, there is some degree of overlap between the two classes.

It is visually obvious that at the two extremes of the range, it is a relatively easy task to predict the correct class for a given feature value, while carrying out the same task in or near the middle of the range is likely to be more challenging or even daunting. Formally speaking, let D be a training dataset of pre-classified instances:

$$D=\left\{\left(x,\;y\right)\in R^{n}\times \left\{\omega_{1},\ldots ,\;\omega_{m}\right\}\right\}$$

where x and *y* denote a feature vector of an input eye status and its true class label, respectively. In the current classification learning problem, the primary goal is to correctly assign the most probable of the available classes $\omega =\{\omega_{1},\ldots ,\;\omega_{m}\}$ for a given eye-status pattern represented by the feature vector $x={\left(x_{1},\ldots ,\;x_{n}\right)}^{⊤}$, where $x_{i}$ is the value of the i-th attribute. For establishing optimal class labeling for unseen eye-status patterns, the maximum a posteriori (MAP) decision criterion is applied to achieve minimal misclassification rate:

(6) $$\omega_{\mathrm{MAP}}^{*}\equiv \underset{\omega_{j}\in \omega }{arg max}\;p\left(\omega_{j}|x\right)$$

The MAP decision rule implies that the feature vector x is assigned to class $\omega^{*}$ where $p\left(\omega^{*}|x\right)>p\left(\omega_{j}|x\right),\;\omega_{j}\neq \omega^{*}\in \omega$. To determine this class, estimates for the conditional probabilities $p(\omega_{j}|x)$ are needed. In order to achieve this objective, we appeal to Bayes’ theorem, which states that:

(7) $$p\left(\omega |x\right)=\frac{p(x|\omega )p(\omega )}{p(x)}$$

where the above probabilities are defined as:

p(ω): independent probability of ω (i.e., prior probability);
p(x): independent probability of x (i.e., evidence);
p(x|ω): conditional probability of x given ω (i.e., likelihood);
p(ω|x): conditional probability of ω given x (i.e., posterior probability).

  Upon applying the Bayes theorem, the MAP class given in Equation (6) can be computed as:

(8) $$\begin{array}{ccc} \omega_{\mathrm{MAP}}^{*} & \equiv & \underset{\omega_{j}\in \omega }{arg max}\;p\left(\omega_{j}|x\right)\\ & = & \underset{\omega_{j}\in \omega }{arg max}\;\frac{p\left(x|\omega_{j}\right)p\left(\omega_{j}\right)}{p(x)}\\ & = & \underset{\omega_{j}\in \omega }{arg max}\;p\left(x|\omega_{j}\right)p\left(\omega_{j}\right) \end{array}$$

Notice that the quantity p(x) is omitted from Equation (8), since the data probability is constant (and independent of class) and thus can be safely excluded from calculations. Now all classes are assumed to be equally probable a priori (i.e., assuming a uniform prior); $p\left(\omega_{j}\right)=p\left(\omega_{k}\right)\;\forall \;\omega_{j},\;\omega_{k}\in \omega$. Consequently, the calculations of the posterior distributions are greatly simplified:

(9) $$\omega_{\mathrm{ML}}^{*}=\underset{\omega_{j}\in \omega }{arg max}\;p\left(x|\omega_{j}\right)$$

In this case, the so-called the maximum likelihood (ML) estimate that maximizes the likelihood of the training data is found. Recalling the MAP rule from Equation (8), we observe that Bayesian classification model depends both on the joint probability and the prior probability:

(10) $$p\left(\omega |x\right)\propto p\left(x|\omega \right)p\left(\omega \right)=p\left(x_{1},\ldots ,\;x_{n}|\omega \right)p\left(\omega \right)$$

Intuitively, the inherent difficulty arising here is involved in learning the joint probability $p(x_{1},\ldots ,\;x_{n}|\omega )$. To overcome this difficulty and make calculations increasingly tractable, we use the well-known "naïve Bayes independence assumption," which states that the probabilities of each attribute are conditionally independent of each other. This allows the joint probability to be conveniently written as a product of conditional probabilities:

(11) $$\begin{array}{ccc} p(x_{1},\ldots ,\;x_{n}|\omega ) & = & p\left(x_{1}|x_{2},\ldots ,\;x_{n};\omega \right)p\left(x_{2}\ldots ,\;x_{n}|\omega \right)\\ & = & \vdots \\ & \approx & p\left(x_{1}|\omega \right)p\left(x_{2}|\omega \right)\ldots p\left(x_{n}|\omega \right)\\ & = & \prod_{i=1}^{n}p\left(x_{i}|\omega \right) \end{array}$$

**Figure 9:**
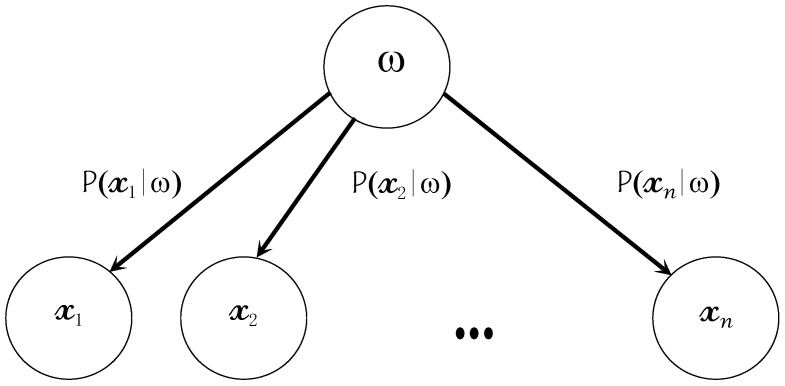
Naïve Bayes model with the assumption of conditional independence.

At this point, it should be pointed out that even though the NB assumption is almost always violated in practice, NB learning is remarkably effective in practice. The substitution of the joint probability from Equation (11) in Equation (8) generates the NB model (depicted in Figure 9),

(12) $$\omega_{NB}=\underset{\omega_{j}\in \omega }{arg max}\left(p\left(\omega_{j}\right)\prod_{i=1}^{n}p\left(x_{i}|\omega_{j}\right)\right)$$

The above conditional probability term in Equation (9) can be estimated as relative frequency, simply by dividing each frequency $n_{c}$ by the total number of opportunities. This approach can lead to significantly poor estimates, even when $n_{c}$ is extremely small. To tackle such a challenging task, the conditional probabilities are estimated as follows:

(13) $$\hat{P}\left(x_{i}|\omega_{j}\right)=\frac{n_{c}+m\alpha }{n+m}$$

where $n_{c}$ and *n* are the number of instances for which $\omega =\omega_{j}$ and $x=x_{i}$, and the total number of training instances for which $\omega =\omega_{j}$, respectively. The parameter α denotes an a priori estimate (in calculations, it is set as $\alpha =\frac{1}{t}$ for *t* possible values of $x_{i}$) and m≥1 is a weight that is specified a priori. With regard to the a priori probabilities, they can in principle be estimated by simply counting the proportion of classes in the training dataset:

(14) $$\hat{P}\left(\omega_{j}\right)=\frac{\#(\omega_{j})}{\sum_{j}\#\left(\omega_{j}\right)}$$

where # denotes the frequency with which a certain class occurs within the available training data. Therefore, the MAP decision rule is equivalently written as:

(15) $$\omega_{NB}=\underset{\omega_{j}\in \omega }{arg max}\left(\hat{P}\left(\omega_{j}\right)\prod_{i=1}^{n}\hat{P}\left(x_{i}|\omega_{j}\right)\right)$$

**Figure 10:**
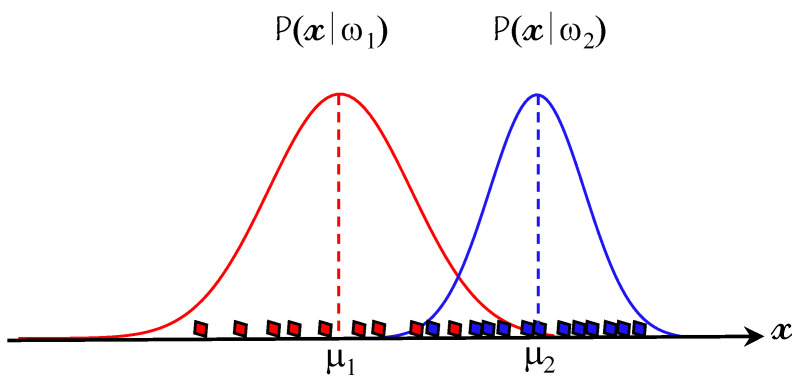
Class-conditional probability distributions of features.

In cases of continuous-valued features, the class-conditional probabilities (likelihoods) p(x|ω) are well-modeled by a Gaussian distribution $N(\mu ,\;\sigma^{2})$:

(16) $$\hat{P}\left(x_{i}|\omega_{j}\right)=\frac{1}{\sqrt{2\pi }\sigma_{ij}}e^{-\frac{1}{2}{\left(\frac{x_{i}-\mu_{ij}}{\sigma_{ij}}\right)}^{2}}$$

where $\mu_{ij}$ and $\sigma_{ij}^{2}$ are the mean and variance of the i-th feature of an eye-state example in the *j*-th class $\omega_{j}$, respectively (see Figure 10).

Based on the above analysis and derivations, the fundamental steps of the NB classification algorithm developed for automated driver drowsiness detection are outlined in Algorithm 1. In regard to the parameter dimensionality of the NB classification model, given a set of *N* data points (or feature vectors of eye-status patterns) and a model with *r* parameters for the probabilities $p(x_{i})$, it is not difficult to discern that the employed NB model will have only a set of mrN+(m−1) parameters, where *m* is the number of classes.
**Algorithm 1:** Naïve Bayes (NB) classification algorithm.
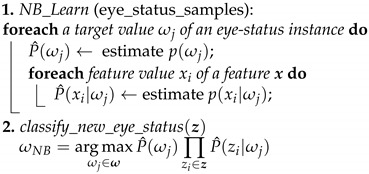


## 3. Experimental Results

In this section, various experimental results are presented and discussed, which are aimed at verifying the superiority of the proposed framework for driver drowsiness detection over competing state-of-the-art approaches in the literature. According to previously reported approaches [32,33,34], there are very few public datasets currently available for comprehensive performance evaluations of different approaches for driver drowsiness detection, particularly those with driver attention information from real-world driving scenarios [35]. On the other hand, it is especially difficult and most dangerous to build a realistic dataset for driver drowsiness detection in real situations that can be used to train the proposed framework comprehensively. For this reason, to verify the effectiveness of the proposed framework for driver drowsiness detection, we conducted extensive experiments on a public dataset, namely, the NTHU Driver Drowsiness Detection (NTHU-DDD) video dataset that is the only publicly-available dataset offering annotations for drowsiness, head, eye-pair and mouth status.

**Figure 11:**
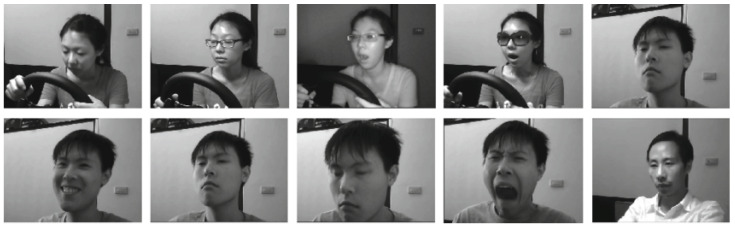
Sample snapshots of the public NTHU-DDD dataset [36].

The academic NTHU-DDD dataset [36] collected by NTHU Computer Vision Lab at National Tsing Hua University was first introduced during the 2016 Asian conference on computer vision (ACCV) on driver drowsiness detection from video. In the dataset collection, the video streams were acquired by a high-speed camera under active infrared (IR) illumination, at a spatial resolution of 640×480 pixels in AVI formate. The total duration of video streams in the entire dataset was almost nine and a half hours. The dataset consists of a total of 36 subjects of various ethnicities who were recorded twice (with and without glasses/sunglasses) under a wide range of challenging simulated driving scenarios, such as normal driving, slow blink rate, yawning, falling asleep and bursting out laughing, under both daytime and nighttime illumination conditions. During video recording, the recruited subjects were asked to sit on a car chair and play a racing game with a simulated driving wheel and pedals; in the meantime they were also asked to perform certain facial expressions. All video sequences were captured in a simulated environment under five different scenarios, namely, "BareFace," "Glasses," "Sunglasses," "Night-BareFace," and "Night-Glasses." The sequences of the first three scenarios have a frame rate of 30 fps, while those of other scenarios have 15 fps. In Figure 11, sample snapshots of NTHU drowsy driver detection (NTHU-DDD) dataset are shown.

In our experiments, the entire NTHU-DDD dataset was split into a training set and an independent test set for the evaluation of the proposed framework. The training set consisted of 356 video samples from 18 subjects, while the test set contained 20 video samples from four subjects. We further divided the training set into videos from four subjects (for validation) and those from the remaining 14 subjects (for training). All selected test videos were resampled to be 15 fps to ensure statistical consistency between training and test data.

In order to quantitatively evaluate the drowsiness detection of the proposed framework, accuracy and F1-score (i.e., the harmonic mean of precision and recall) were calculated for each simulated driving scenario, where precision and recall are defined as follows:

(17) $$\begin{array}{ccc} precision & = & \frac{TP}{TP+FP}\\ recall & = & \frac{TP}{TP+FN}\\ F1-score & = & 2\times \frac{precision\times recall}{precision+recall} \end{array}$$

where TP (true positive) is the number of the correct drowsiness predictions, FP (false positive) is the number of incorrect drowsiness predictions (type I errors), TN (true negative) is the number of correct non-drowsiness predictions and FN (false negative) is the number of incorrect non-drowsiness predictions (type II errors). Detailed detection results of the proposed framework in terms of accuracy and F1-score for each simulated driving scenario in the NTHU-DDD dataset are provided in Table 1.

From the results shown in the above table, the following interesting observations can be made. First, perhaps the most remarkable fact emerging from the table is that the proposed framework achieved an average accuracy of 85.62% for drowsiness detection, which is very encouraging and agrees well with prior results reported in the literature. Furthermore, in light of these results, one can argue that a high level of accuracy in the drowsiness detection task along with significantly low computational costs would greatly contribute to the feasibility and robustness of the proposed framework for real-time traffic monitoring. Additionally, in order to assess the competitive performance of the proposed approach, we provide an experimental performance comparison of the framework against several state-of-the-art methods [17,37,38,39,40] in terms of accuracy of drowsiness detection. Table 2 provides a summary of this comparison. In light of the comparison, it is contended that the proposed framework exhibits superior results compared with other state-of-the-art approaches, while guaranteeing the deadline of the real-time traffic monitoring. In that vein, it is also worth mentioning that all the methods considered in the above comparison (Table 2) used the same dataset and almost similar experimental setups. Therefore, the comparison is apt to be most valid and revealing.

In closing, we thus conclude that the experimental results have shown convincing evidence that the proposed system has the potential to improve the performance of driver drowsiness detection systems, without loosing the real-time guarantee; the system is capable of maintaining real-time processing of 24 fps. As a final concluding point, it is also worthwhile to mention that all the algorithms in this framework were implemented in Microsoft Visual Studio 2015 with OpenCV vision library version 4.1.2 for the graphical processing functions. All experiments (including tests and evaluations) were conducted on a PC with an Intel(R) Core(TM) i7 CPU-3.07 GHz processor, 8GB RAM, running Windows 10 Professional 64-bit operating system. As it might be expected, the achieved results demonstrate that the presented system can operate reliably and efficiently, achieving real-time performance operation for video sequences, due to the use of highly efficient algorithmic implementations in OpenCV library in combination with custom C++ functions.

## 4. Discussion and Conclusions

This paper has proposed an effective framework for instantaneous driver drowsiness detection, using an adaptive variant of HOG features for eye region representation and an NB model for classification. Quantitative evaluations on the publicly-available NTHU-DDD dataset have consistently demonstrated not only the significant superiority of the proposed system over several recent state-of-the-arts, but also its ability to make real-time predictions about whether drivers are drowsy or fatigued. A possible limitation of the framework relates to the generalizability of drowsiness detectors that have been developed based on the NTHU dataset, since video footage obtained in a moving car, in realistic traffic scenes, with sleepy drivers, is an entirely different thing than people in a laboratory who act sleepy or fatigued (as in the case of the NHTO dataset). We suspect that an additional limitation might arise from neglecting the effect of repetitive ambient change during driving and its effect of drowsiness detection rate in experiments. As prospects for future work, the intentions are twofold. On the one hand, we aim at enhancing the validity and applicability of the presented methodology and extending implementations to integrate more diverse datasets to investigate the scalability of the proposed framework. On the other hand, we intend to extend the basic architecture of the framework for use in embedded boards or microcomputing systems to reduce operating and financial expenses and improve computational costs, without noteworthy performance degradation.

## Figures and Tables

**Table 1:** Detailed results of the detection performance of the proposed framework on the public NTHU-DDD dataset.

Scenario	Drowsiness F1-Score (%)	Non-Drowsiness F1-Score(%)	HOG AC (%)	advHOG AC (%)
Bareface	90.99	87.19	86.21	89.35
Glasses	85.07	72.10	78.36	80.31
Sunglasses	81.74	67.14	75.69	76.30
Night-BareFace	93.49	85.37	87.64	90.64
Night-Glasses	87.93	93.68	88.24	91.48
Average	87.84	81.09	83.19	85.62

**Table 2:** Quantitative comparison with other recent state-of-the-arts on the NTHU dataset

Method	Accuracy (%)
Proposed Method	85.62
CNN-LSTM [41]	75.80
Scale-Pruned 3D-CNN [39]	78.48
seqMT-DMF [37]	83.44
MSTN [38]	82.61
Joint-Shape RF [42]	88.18
3D-DCNN [18]	87.46
Human [17]	80.83

## Data Availability

Data sharing is not applicable to this article.
